# State-of-the-art silicone molded models for simulation of arterial switch operation: Innovation with parting-and-assembly strategy

**DOI:** 10.1016/j.xjtc.2021.12.009

**Published:** 2022-01-19

**Authors:** Brandon Peel, Whal Lee, Nabil Hussein, Shi-Joon Yoo

**Affiliations:** a Center for Image-Guided Innovation and Therapeutic Intervention, The Hospital for Sick Children, University of Toronto, Toronto, Ontario, Canada; b Department of Radiology, Seoul National University Hospital, Seoul, Korea; c Department of Congenital Cardiac Surgery, Yorkshire Heart Centre, Leeds General Infirmary, Leeds, United Kingdom; d Department of Diagnostic Imaging, The Hospital for Sick Children, University of Toronto, Toronto, Ontario, Canada; e Division of Cardiology, Department of Paediatrics, The Hospital for Sick Children, University of Toronto, Toronto, Ontario, Canada

**Keywords:** three-dimensional printing, silicone molding, congenital heart surgery, simulation, hands-on surgical training, arterial switch operation, transposition of the great arteries, 3D, three-dimensional, ASO, arterial switch operation, CHS, congenital heart surgery, HOST, Hands-On Surgical Training, TGA, transposition of the great arteries

## Abstract

**Background:**

Three-dimensional (3D) printed models are widely accepted for use in training of various surgical procedures for congenital heart disease; however, their physical properties have been considered suboptimum for procedures. We created silicone molded models produced using a novel “parting and assembly” strategy and compared their suitability for hands-on training with that of conventional 3D printed models.

**Methods:**

Computed tomography imaging data from 2 patients with transposition of the great arteries were used. The heart was divided into multiple parts (atria, ventricles, great arteries, coronary arteries, and valves), and molds of each part were created. The parts reproduced by silicone molding were assembled using an adhesive agent. In an online course, 2 silicone molded models and 1 3D printed model were used for training of 34 surgeons. A questionnaire was distributed to these surgeons aimed at assessing the suitability of the models for the arterial switch operation (ASO).

**Results:**

The silicone molded models showed excellent anatomic detail, high elasticity, and high resistance to tearing. The cost per model, based on the production of 50 models, was slightly higher for the silicone molded models compared with the 3D printed models. All 26 surgeons who completed the questionnaire reported that the silicone molded models provided sufficient anatomic information, but only 19% said the same for the 3D printed models. All surgeons also considered the silicone models to be realistic when passing a needle, cutting vessels, suturing, and excision of the coronary buttons, as opposed to <46% for the 3D printed models.

**Conclusions:**

Silicone molding of models for the ASO is feasible by applying a “parting and assembly” strategy. Silicone molded models provide excellent physical properties that are far superior to those of 3D printed models for surgical simulation.

**Figure:**
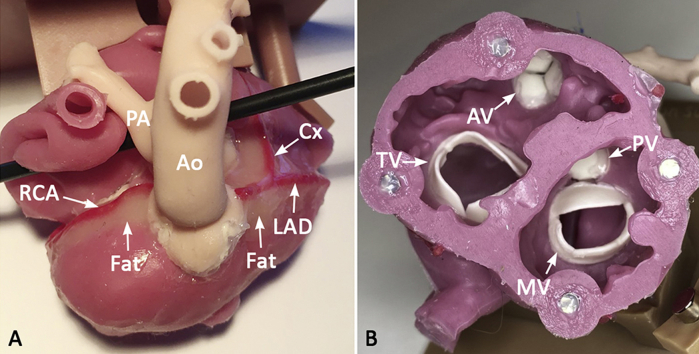
Exterior and interior of a silicone molded model of transposition of the great arteries for the arterial switch operation. *Ao*, aorta; *AV*, aortic valve; *Cx*, circumflex coronary artery; *LAD*, left anterior descending coronary artery; *MV*, mitral valve; *PA*, pulmonary artery; *PV*, pulmonary valve; *RCA*, right coronary artery; *TV*, tricuspid valve.

**Central Message**

Silicone molding with a “parting and assembly” strategy allows modeling of high-fidelity surgical simulation models with high elasticity and resistance to tear for the arterial switch operation.

**Perspective**

Silicone molded models are overwhelmingly superior to conventional 3-dimensional (3D)-printed models for surgical simulation training. The high elasticity, resistance to tear, and multiple color options of silicone molded models enable realistic simulation of the arterial switch operation. The concept can further be applied to other pathologies, such as septal defects and valvular abnormalities.

There is a growing expectation of perfect patient outcomes in congenital heart surgery (CHS), which has emphasized the need for improved methods of surgical training.^1^, ^2^, ^3^, ^4^ Hands-on simulation has been suggested as an important adjunct in the development and improvement of surgical skills,^3^, ^4^, ^5^ with 3D printed models providing excellent resources for simulation of these complex procedures.^6^, ^7^, ^8^ Since its introduction in 2015, Hands-On Surgical Training (HOST) courses using 3D printed models have received excellent responses, which has driven regular annual courses and its incorporation into monthly surgical training curricula and virtual formats.^6^, ^7^, ^8^, ^9^, ^10^, ^11^ However, commercially available materials for 3D printing provide suboptimal elastic properties, strength, and texture for surgical procedures.^6^^,^^9^^,^^10^^,^^12^, ^13^, ^14^, ^15^ Silicone has been used for variety of medical applications because its physical properties are similar to those of human soft tissues.^16^ Despite the growing demand for innovation, current 3D printing technology for silicone modeling is considered inadequate for the production of complex congenital heart disease models. Alternatively, the injection molding technique has been used for fabrication of silicone-based simulation models.^17^, ^18^, ^19^ However, the complexity of the cardiovascular anatomy has limited use of the silicone molding technique to modeling relatively simple structures, such as cardiac valves and vascular structures.^17^, ^18^, ^19^, ^20^

Here we describe the development and integration of silicone molded hearts into the HOST simulation program. We hypothesized that a silicone molded heart model of transposition of the great arteries (TGA) can be created using an injection molding technique with a “parting-and-assembly” strategy whereby the heart is divided into multiple parts and assembled using an adhesive agent. We also hypothesized that silicone molded models provide a superior and more realistic congenital heart surgery (CHS) experience than the best currently available 3D printed materials.

## Methods

### Segmentation and Computer-Aided Design Process

**Figure 1:**
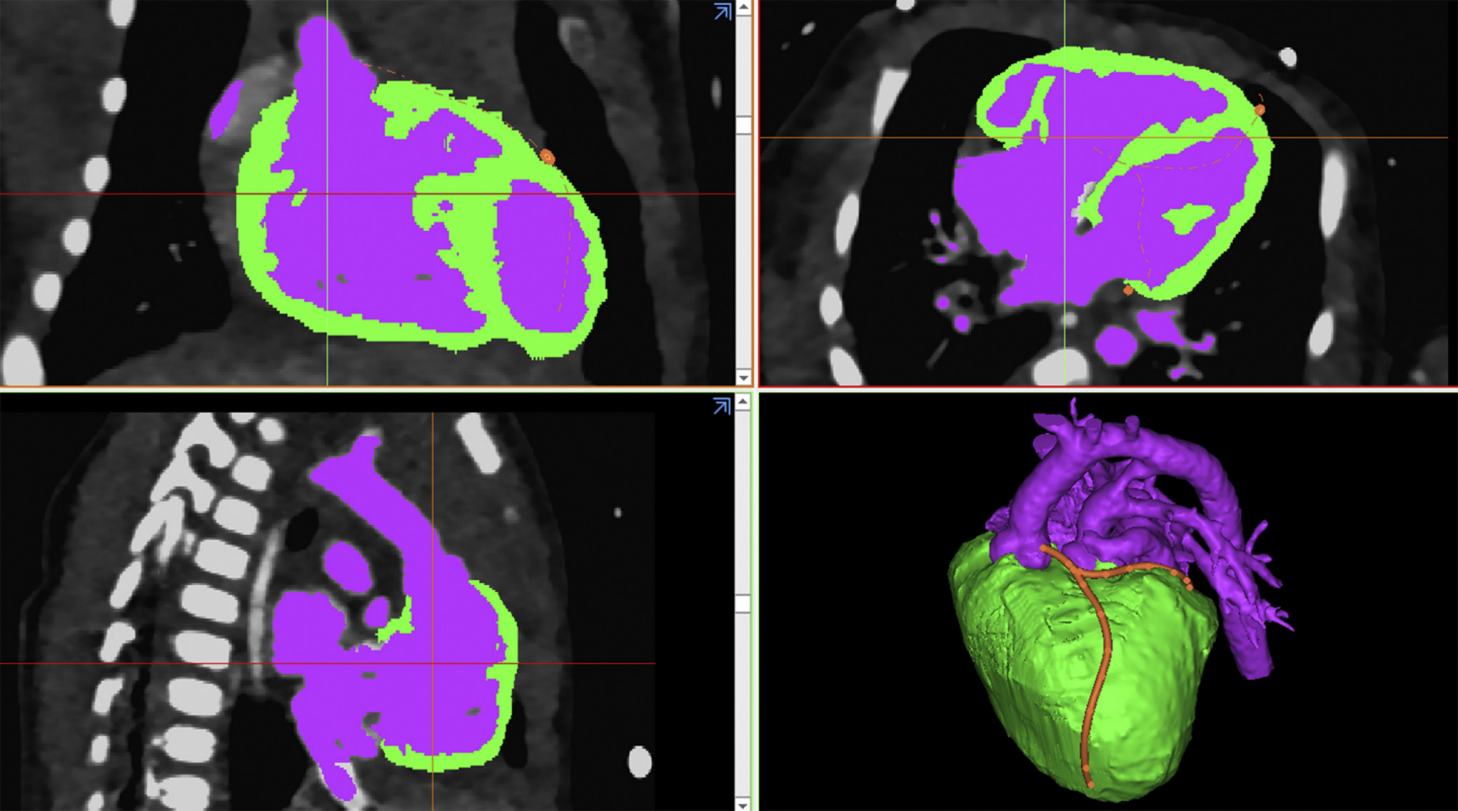
Postprocessing panels showing segmentation of the blood pool (*purple*) and the ventricular myocardium (*green*). The coronary artery route is traced in red as shown in the bottom right panel.

**Figure 2:**
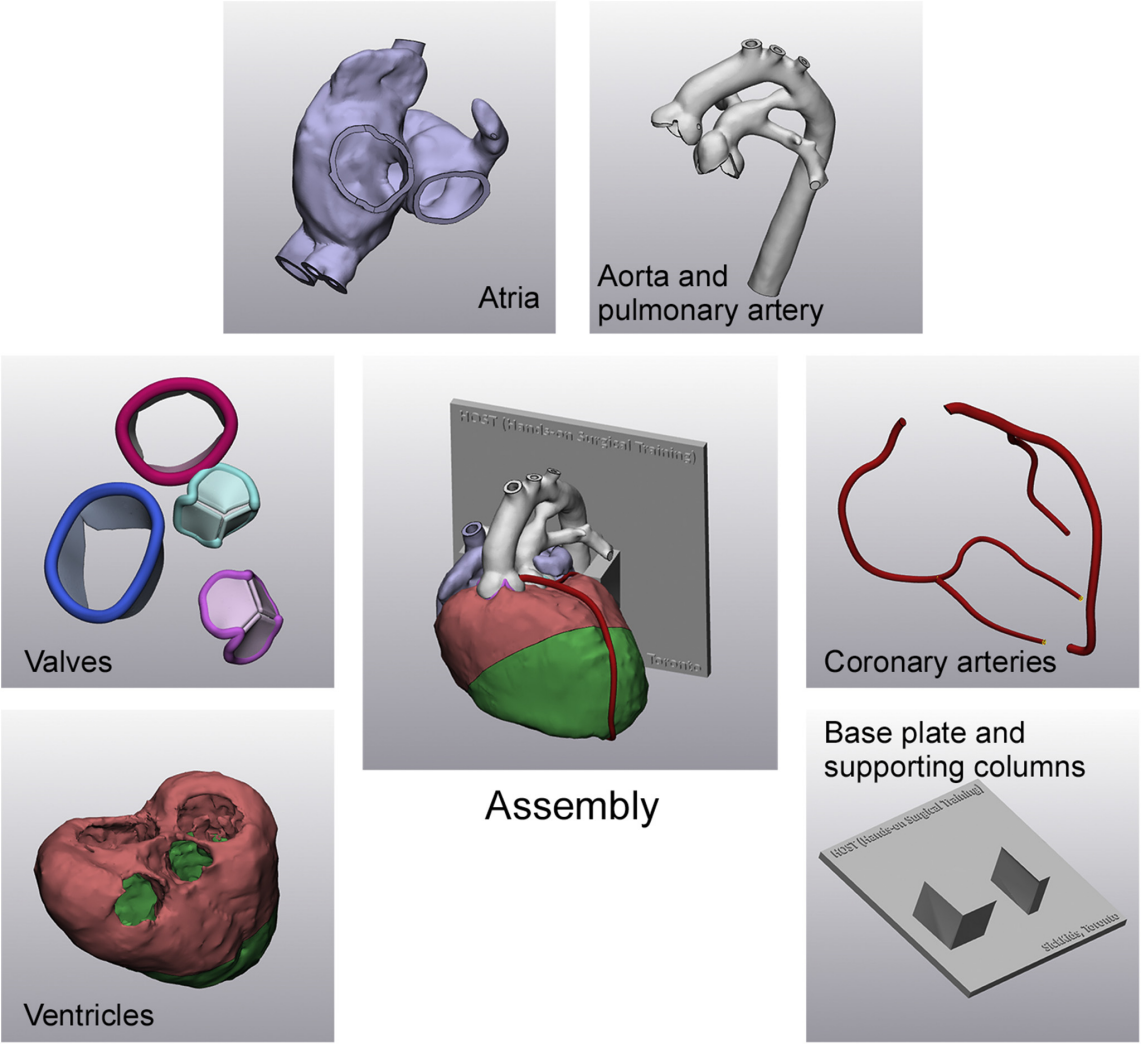
Individual parts used to develop the transposition of the great arteries model for simulation of the arterial switch operation. The center panel shows the complete assembly of the parts.

Two electrocardiographically gated computed tomography scans from 2 newborn infants with TGA were used. Segmentation of the DICOM images and computer-aided design processes were performed using commercially available software programs (Mimics Medical 23.0 and 3-Matic Medical 15.0; Materialise NV) as described elsewhere (Figure 1).^21^ Because it was not possible to apply the molding technique to form a single integrated piece without sacrificing the mold during removal of the model, the object was divided into multiple parts, including the atria, ventricles, pulmonary artery, aorta, coronary arteries, and cardiac valves (Figure 2). The ventricular myocardium was directly segmented with manual editing (Figure 1). Because direct segmentation of the atrial and vessel walls was not possible, graphically designed walls were added to the surface of the blood pool. The atrial wall was created by hollowing the blood pool with a 0.6 mm thickness in both directions, resulting in a total thickness of 1.2 mm. Vascular walls were created by hollowing the blood pool inward with a 0.9 mm thickness. The attachment sites of the cardiac valves and the expected free edges of the leaflets were traced with a spline tool on the original segmented model, and the valve leaflets were graphically designed.^21^

Tracing of the valve leaflets involved the operator's knowledge-based approximation of the leaflet configuration. Once the edge was traced, the valve was designed by reconstructing a surface defined by the traced edge and adding a 0.4-mm-thick wrap around the reconstructed surface. The positions of the valve leaflets were simulated for diastole (ie, in the open position for the tricuspid and mitral valves and the closed position for the aortic and pulmonary valves). The major epicardial coronary arteries were traced using the ‘sweep-loft’ function and integrated into the model. All separate parts of the model were saved in STL file format, and prototypes were printed on a 3D printer (J750 Digital Anatomy 3D Printer; Stratasys) using photopolymer resins (Agilus for atrial and vessel walls and Digital Anatomy TissueMatrix [Stratasys] for ventricular myocardium).

### Silicone Molding

**Figure 3:**
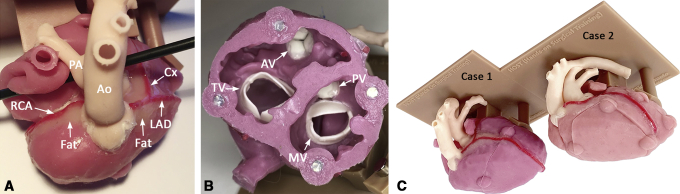
Assembled silicone molded model used for simulation of the arterial switch operation. A, Superior aspect of the model showing the typical anterior-posterior relationship between the ascending aorta (*Ao*) and the main pulmonary artery (*PA*). The right coronary artery (*RCA*) and left coronary artery arise from their respective sinuses. The space around the coronary arteries is filled with beige-colored silicone to simulate epicardial fat. B, Basal view of the ventricles looking from the cardiac apex, showing all the cardiac valves. C, Completed models mounted on a basal plate and supporting columns. *AV*, Aortic valve; *Cx*, circumflex coronary artery; *LAD*, left anterior descending coronary artery; *TV*, tricuspid valve; *MV*, mitral valve; *PV*, pulmonary valve.

**Figure E1:**
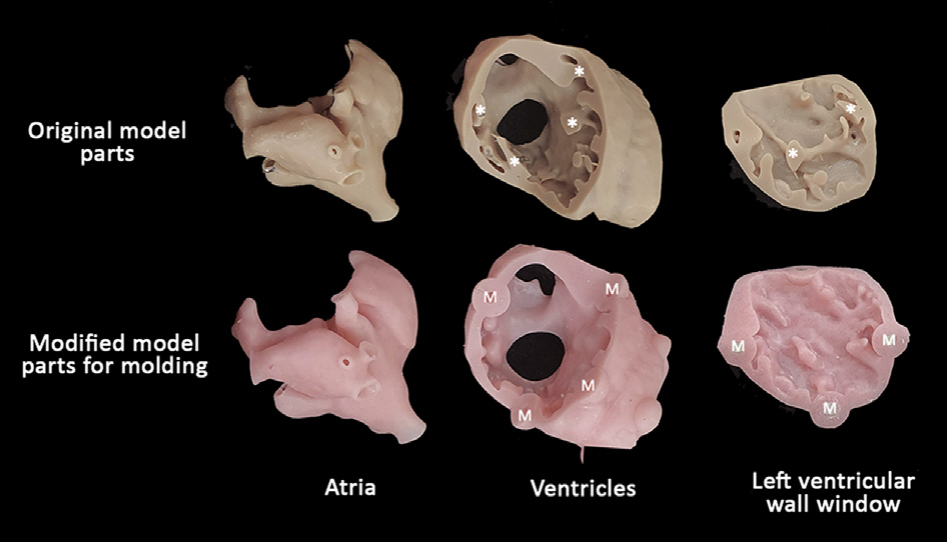
The original 3-dimensional printed model parts and the modified model parts used for molding. Note that the muscle bundles (*asterisks*) in the ventricles are abbreviated in the modified model parts, whereas the atrial structures are almost identical in both the original and modified model parts. Magnets (M) were added in the modified model to secure the left ventricular wall window to the model.

The STL files and 3D printed models were reviewed for applicability of the molding technique. For structures that could not be reproduced by molding, the STL files were further modified, and structures of little surgical importance (eg muscle bundles crossing the RV cavity) were transected or removed (Figure E1). When muscle bundles needed to be preserved, they were divided and later reattached during the assembly stage. Silicone fluids of various colors with a shore hardness value were infused into the molds. The silicone casts produced were assembled using an adhesive agent by closely referencing the digital files displayed on a computer screen. The spaces around the coronary arteries were filled with light-beige silicone (shore hardness value of 0.1) to simulate epicardial fat surrounding the coronary arteries. Finally, the models were mounted on a 3D printed basal plate with supporting columns (Figure 3). The molding process was outsourced to a commercial company (Gluck Co Ltd).

### The 2020 HOST Course Format

**Figure 4:**
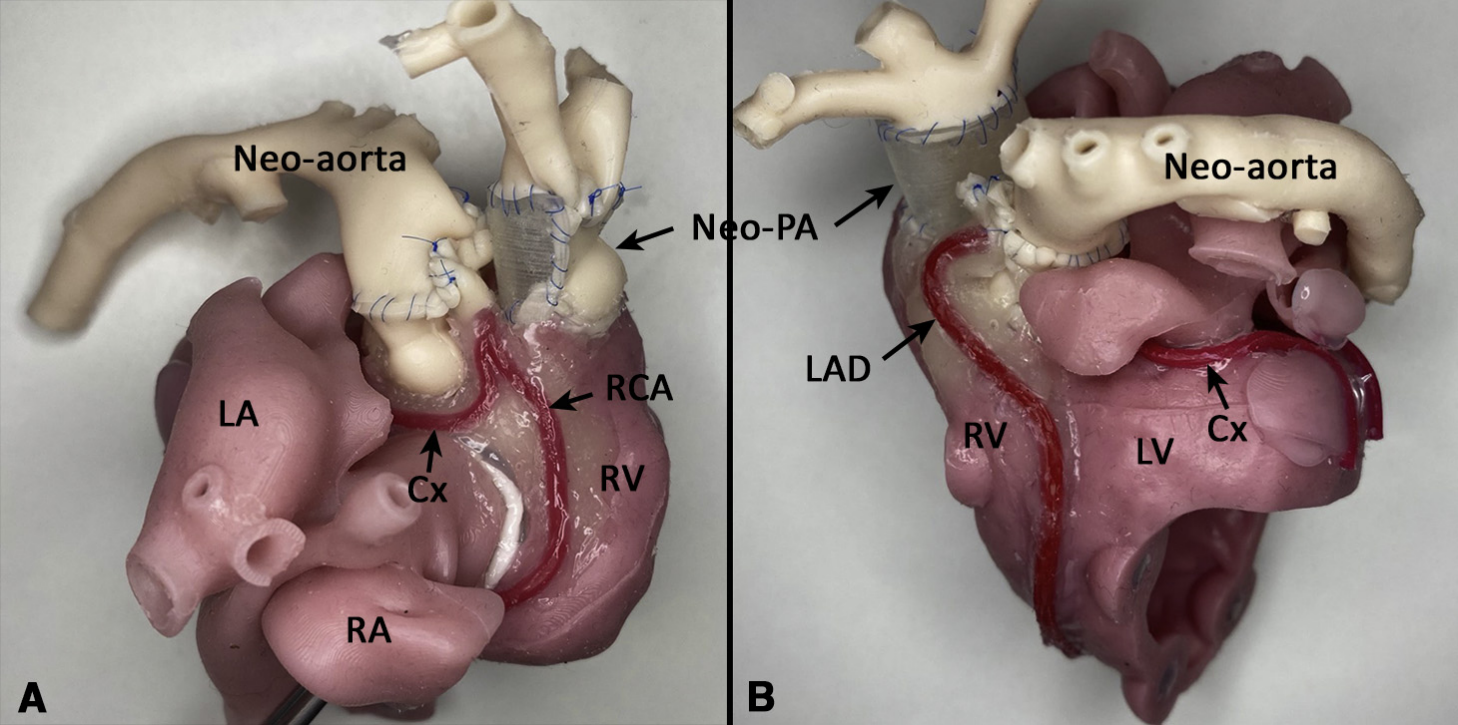
Right (A) and left (B) anterior oblique views of the model after simulation of the arterial switch operation performed by a training surgeon as part of the Hands-On Surgical Training course. The neo-aorta is now posterior, arising from the left ventricle *(LV*) with the reconstructed neo-pulmonary artery (neo-PA) arising from the right ventricle (*RV*). Note how the coronary arteries have been reimplanted on the neo-aorta. *LA*, Left atrium; *RA*, right atrium; *Cx*, circumflex artery; *RCA*, right coronary artery; *LAD*, left anterior descending artery.

The silicone TGA models for the arterial switch operation (ASO) were used at the annual HOST course organized by the Hospital for Sick Children, Canada in 2020, with 34 cardiovascular surgeons in attendance. The surgeons’ experience level ranged from resident to staff pediatric surgeon (Table E1). Each participant received 3 different surgical simulation models demonstrating different variations of the disease. One of the models was directly 3D printed as used in previous HOST courses,^9^ and the other 2 models were the new silicone molded models. After observing a demonstration of the ASO on the model by an experienced surgeon, each attendee simulated the same procedure while proctors provided technical advice and feedback on performance (Figure 4). On course completion, a questionnaire was distributed to the attendees to rank the suitability of the models for the ASO simulation.

## Results

The estimated production times and costs of the 2 types of models are summarized in Tables 1 and 2. Silicone molding is labor-intensive and requires additional segmentation, computer-aided design, the production of multiple molds, and silicone molding. For the production of 50 models, silicone molding requires a slightly longer production time and higher cost than conventional 3D printing. The advantages and disadvantages of 3D printed and silicone molded models are summarized in Table 3. The silicone molded models provide excellent physical properties for surgical simulation and are extremely resistant to traction forces, whereas the 3D printed models can be easily torn with increased physical force during such steps as suturing. The multiple color options used in silicone molding allow for ready perception of the boundaries of the cardiac valves and coronary arteries, along with better overall esthetics and realism. The major disadvantage of the silicone molding technique is limitations in the accurate representation of the hanging muscle bundles within the cardiac cavity, which is of no significance in the ASO.

**Table 1:** Estimated time required for production of 50 models by conventional 3D printing and silicone molding

Production step	3D printing	Silicone molding
Segmentation of image data	8 h total; 0.15 h per model	12 h total; 0.24 h per model
Computer-aided design	5 h total; 0.1 h per model	10 h total; 0.2 h per model
Creation of silicone molds	—	50 h total; 1 h per model
3D printing (include cleaning and coloring) per model	10 h	15 h total; 0.3 h per model
Silicone molding and assembly per model	—	10 h
Total per model	10.25 h	11.74 h

*3D*, 3-dimensional.

**Table 2:** Production parameters for 3D printed and silicone molded models

Parameter	3D printed models	Silicone molded models
Production process	Relatively simple	Complicated
Production time excluding segmentation and CAD	10 h per model by any operator without supervision	11.3 h per model (estimated for production of 50 models) by trained and experienced operators
Required workforce after segmentation and CAD	Low	Labor-intensive
Typical production cost per model	300 USD	350 USD (estimated for production of 50 models)

*3D*, 3-dimensional; *CAD*, computer-aided design.

**Table 3:** Advantages and disadvantages of 3D printed and silicone molded models based on authors' subjective assessment and surgeons’ questionnaire responses

	3D printed models	Silicone molded models
Advantages	• Accurate representation of anatomic detail • Straightforward production process after segmentation and computer- aided design • Small workforce required • Can produce any number of models	• Physical properties closer to human tissue • High elasticity and resistance to physical force during traction and suturing • Ample choice of silicone materials with different physical properties and colors • No adherence between adjacent structures • No significant degradation of model quality with time
Disadvantages	• Limited availability of printing materials with different physical properties • Low elasticity and resistance to physical force during traction and suturing • Adherence between adjacent structures • Different color options available at the cost of compromised softness and elasticity • Degradation of model quality over time	• Limitation in representation of hanging structures such as trabeculations • Additional segmentation and computer-aided design • Labor-intensive production process for silicone molding and assembly of parts • Not applicable for a small number of models due to a high cost • Logistical difficulty in on-site production
Cost	• Equal production cost after segmentation and computer aided design; little advantage of high-volume production	• High cost for low-volume production and reduced cost for high-volume production

*3D*, 3-dimensional.

**Figure E2:**
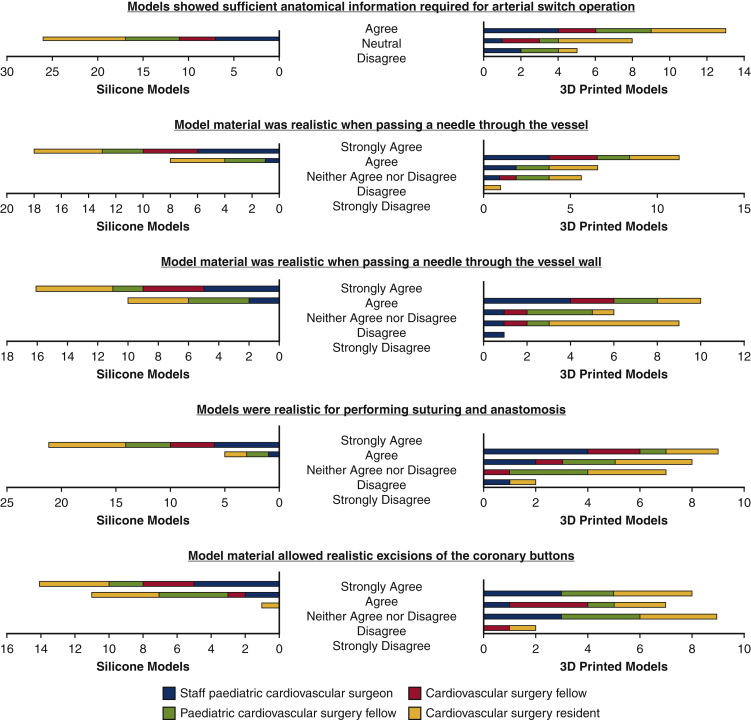

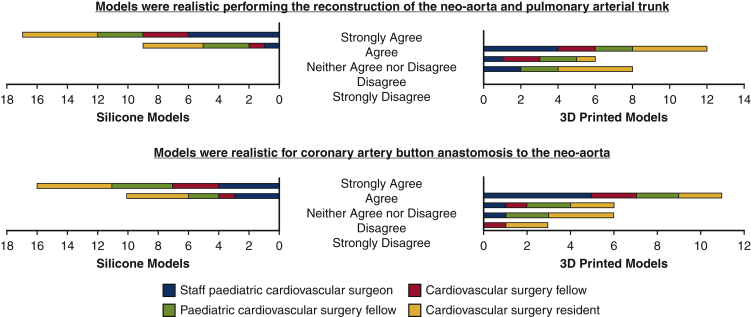
Questionnaire responses from 26 surgeons who attended the 2020 annual Hands-On Surgical Training course on the arterial switch operation comparing the new silicone models and conventional 3-dimensional printed models.

All 34 participating surgeons completed the surgical simulation procedures on the 3 models with the proctors' online supervision. Twenty-six surgeons (76%) completed the postcourse questionnaire (Figure E2). All respondents agreed that the silicone molded models provided sufficient anatomic information for simulation, whereas only 5 respondents (19%) believed the same about the 3D printed models. The silicone models performed better, with all surgeons agreeing that the material was realistic for general tasks, such as passing a needle through tissues, cutting vessels, and suturing, and allowed for realistic excision of the coronary buttons, compared with <46% who believed the same for the 3D printed model. Furthermore, all surgeons agreed that the silicone models were realistic for performing key operative tasks, including reconstruction of the neo-aorta/pulmonary arterial trunk and coronary artery button anastomosis, whereas <50% believed the same for the 3D printed models. Respondents’ additional comments on the course included the general advantages as well as disadvantages of the online course and the need for an assistant, a structured program with progression from simpler to complex procedures, and detailed information on the simulation setup, including the size of the models and the required needles (Video Abstract).

## Discussion

3D printed modeling has enabled the growth of HOST for simulation of CHS procedures.^6^, ^7^, ^8^, ^9^, ^10^, ^11^ Although they have been well received by users, the 3D printed models produced with the currently available technology do not have the optimal physical properties for traction, cutting, and suturing during simulation.^6^^,^^9^^,^^10^^,^^12^, ^13^, ^14^, ^15^ To overcome the weaknesses of the 3D printed models, silicone molded models of TGA for ASO were successfully introduced at the 2020 Annual HOST course. The major breakthrough in silicone molding in this study was the “parting and assembly” strategy, in which the separate components of the model are created using different silicone thickness, shore hardness, and colors and are manually assembled. This strategy allows molding of complex structures, the use of multiple realistic colors for better differentiation of the model parts, and modeling without adhesion between the structures in direct contact, all of which are limitations of the traditional single-piece molding technique as well as of 3D printing.

All surgeons who completed the questionnaire noted that the silicone material allowed for a superior simulation experience for various surgical steps in ASO compared with the best currently available 3D printed models. The surgeons noted that the silicone molded models provided a close anatomic similarity to reality. Most importantly, the silicone molded models provided excellent elasticity and resistance to tear by the force of traction and suturing. As opposed to the uniform color of the 3D printed models, in the silicone molded models, different components can be produced in different colors without compromising the material properties, which helps to reproduce the intraoperative reality and aids the simulation.

Although the number of trainees who attended the course and provided feedback was small, the overall consensus from the study participants strongly suggests that the incorporation of silicone molded models for CHS is beneficial for the entire simulation experience. Interestingly, throughout our experience in HOST CHS simulation, we have noticed a change in user feedback, with surgeons who were once highly complementary of the 3D printed material now critical of its suitability for surgical simulation.^6^ This shift demonstrates an evolution in user perception as improved materials and better models are produced, which is a testament to developers in the field who acted on feedback and strived to develop higher-fidelity models to improve training further.

The major limitation of silicone molding with the “parting and assembly” strategy is the high production cost, which may exceed that of 3D printing. The assembly is a challenging process that requires delicate hands. The cost-effectiveness of the production line depends on not only the number of tasks involved, but also on the number of models required. The cost of silicone molding will be reduced exponentially with the increasing number of models produced. In the present study, the silicone molding needed to be outsourced not only because of the authors’ lack of experience, but also because of the low cost-effectiveness of in-house production. This is in contrast to 3D printing, in which the production cost remains the same regardless of the number of models produced. Therefore, silicone molding is applicable for the production of a large number of models. Based on our limited experience with TGA models, it becomes cost-effective when more than 50 models are produced. Although the models introduced in this study were designed to show the anatomy of the whole heart, simplified, lower-cost versions can be developed by reduced the detail in the regions of little surgical interest; for instance, the cost of the simulation models for the ASO could be reduced by not including the tricuspid and mitral valve leaflets and the apical halves of the ventricles in the model.

One may consider using the silicone molding technique for producing patient-specific models. However, this would be applicable only for elective surgical procedures at a significantly high cost and would be challenging to apply for urgent procedures. In the Hospital for Sick Children, Canada, we use 3D printing to prototype models for all clinically indicated cases.

The silicone molding technique should be developed further for the reproduction of complex structures such as cardiac valves and supporting structures including chordae tendineae, which will provide more realistic models for closure of ventricular septal defects with tricuspid valve leaflets and chords overlying the defect margin and for cardiac valve repairs such as cone surgery for Ebstein's anomaly. Further characterization of tissue properties is needed to develop silicone materials that closely mimic various parts of the cardiovascular system. Direct 3D printing with silicone has been explored in recent years, but various limitations inhibit the production of models suitable for the simulation of complex anatomy.^16^^,^^22^

Conversely, much work is needed to improve the best commercially available 3D printing materials to withstand the future of surgical simulation. Severseike and colleagues^13^ found that Digital Anatomy materials (Stratasys) more closely resembled the compliance of real tissue compared with other 3D printed materials but did not perform well in terms of suturing and cutting properties. Kwon and colleagues^12^ have proposed a new method for mimicking the mechanical characteristics of the aortic wall using multimaterial 3D printing with embedded patterns. Specifically, they successfully replicated the tensile strength and strain of the aorta, providing promising results that can be used for more realistic surgical training models for commercially available 3D printing materials.

## Conclusions

High-fidelity surgical simulation models for ASO have been reproduced using our “parting and assembly” strategy. Although the production process is laborious, silicone molded models provide accurate anatomic models of congenital heart disease and allow for realistic surgical procedures owing to their high elasticity and resistance to tear. The use of silicone molded models for simulation of the ASO in the annual HOST course proved to be more beneficial for all levels of cardiac surgeons compared with the best currently available 3D printing materials and technology. The inclusion of silicone molded models that mimic both the anatomic reality and mechanical behavior of cardiac tissue into HOST programs for CHS will surpass traditional training on 3D printed models and continue to improve the acquisition of technical surgical skills in cardiovascular surgery trainees.

### Conflict of Interest Statement

The authors reported no conflicts of interest.

The *Journal* policy requires editors and reviewers to disclose conflicts of interest and to decline handling or reviewing manuscripts for which they may have a conflict of interest. The editors and reviewers of this article have no conflicts of interest.

## Figures and Tables

**Table E1:** Participants in the online Hands-On Surgical Training course by experience level.

Participants	Number
Staff pediatric cardiovascular surgeon	7
Pediatric cardiovascular surgery fellow	6
Cardiovascular surgery fellow	4
Thoracic cardiovascular surgery residents	9

## Supplemental Data

Video Abstract
